# Excessive flexed position of the femoral component causes abnormal kinematics and joint contact/ ligament forces in total knee arthroplasty

**DOI:** 10.1038/s41598-023-33183-2

**Published:** 2023-04-19

**Authors:** Kohei Nishitani, Shinichi Kuriyama, Shinichiro Nakamura, Young Dong Song, Yugo Morita, Hiromu Ito, Shuichi Matsuda

**Affiliations:** Department of Orthopaedic Surgery, Graduate School of Medicine, Kyoto University, 54 Shogoin-Kawahara-Cho, Sakyo-Ku, Kyoto, 606-8507 Japan

**Keywords:** Musculoskeletal system, Musculoskeletal system, Osteoarthritis

## Abstract

Poor clinical outcomes are reported in excessive flexion of the femoral component in total knee arthroplasty (TKA), but their mechanisms have not yet been elucidated. This study aimed to investigate the biomechanical effect of flexion of the femoral component. Cruciate-substituting (CS) and posterior-stabilised (PS) TKA were reproduced in a computer simulation. The femoral component was then flexed from 0° to 10° with anterior reference, keeping the implant size and the extension gap. Knee kinematics, joint contact, and ligament forces were evaluated in deep-knee-bend activity. When the femoral component was flexed 10° in CS TKA, paradoxical anterior translation of the medial compartment was observed at mid-flexion. The PS implant was best stabilised with a 4° flexion model in mid-flexion range. The medial compartment contact force and the medial collateral ligament (MCL) force increased with the flexion of the implant. There were no remarkable changes in the patellofemoral contact force or quadriceps in either implant. In conclusions, excessive flexion of the femoral component yielded abnormal kinematics and contact/ligament forces. Avoiding excessive flexion and maintaining mild flexion of the femoral component would provide better kinematics and biomechanical effects in CS and PS TKA.

## Introduction

Total knee arthroplasty (TKA) has successfully improved the quality of life and daily activities of patients with end-stage knee arthritis^1^. Many factors can contribute to clinical outcomes, such as patient condition, implant design, and surgical technique^1–3^. Among surgical techniques, appropriate implant position is one of the key factors for the success of TKA^4,5^.

Although the optimal prosthetic alignment in the sagittal plane is unknown, a slightly flexed position of the femoral component has been recommended^5,6^. Extension of the femoral component can cause anterior femoral notching and may increase the patellofemoral contact pressure^7,8^. To avoid notching, the femoral component can be controlled to make the anterior flange nearly parallel to the anterior cortex of the femur^9^, and a slightly flexed position has been used in navigated TKA^7,10^. However, flexion of the femoral component increases the posterior condylar offset, which can affect knee kinematics and joint tightness^11,12^. A previous report showed that an increase of 2° in the sagittal flexion of the femoral component led to a decrease of 1 mm in the flexion gap^11^. Furthermore, both excessively extended and flexed positions are considered to overstress the polyethylene insert^13,14^.

In a computer simulation study, flexion of the femoral component with a posterior reference has been reported to improve kinematics and biomechanical effects in TKA^8,15^. However, in a previous study, excessive flexion was reported to yield inferior satisfaction and function^16^. In this study, a computer simulation study was employed to investigate the effect of flexion of the femoral component with an anterior reference on knee biomechanics. The hypothesis was that slight flexion of the femoral component would not affect kinematics and joint/ligament force; however, excessive flexion of the femoral component would show abnormal kinematics and/or abnormal joint/ligament force.

## Methods

The present study was approved by Ethics Committee Graduate School and Faculty of Medicine Kymakioto University (registration number R0980) and was performed in accordance with the national Ethical Guidelines for Medical and Health Research Involving Human Subjects and ethical standards in the Declaration of Helsinki. The single participant, with whom the bone model was created, was provided informed consent for risk of this examination, including radiation exposure, and consented.

### Computer simulation model

**Figure 1:**
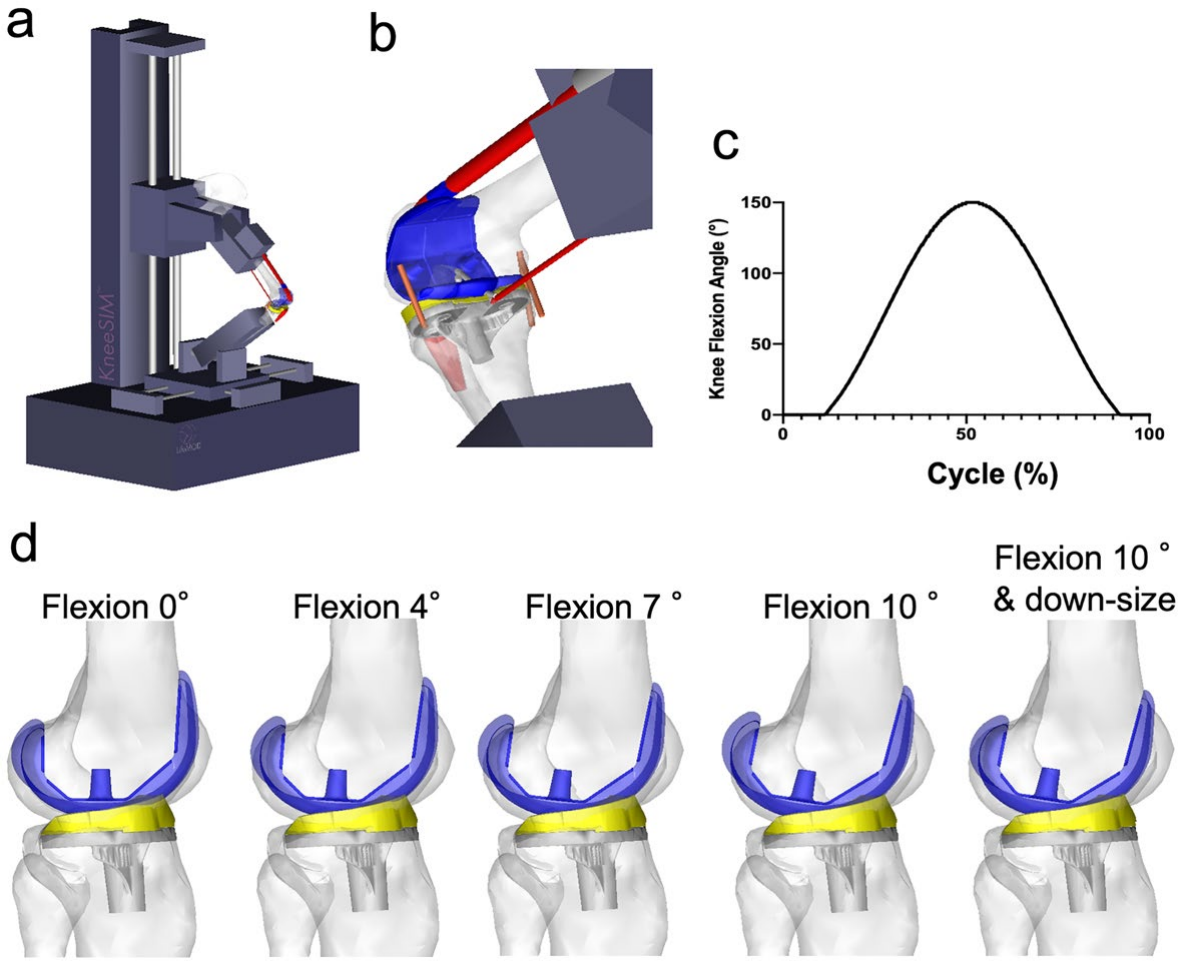
Computer-rendered images. (**a**) Overall picture of this study using Oxford-knee-rig. (**b**) Attachment of ligaments and boundary conditions with the Bi-Surface implant used in this study. (**c**) Simulation protocol and range of motion for deep knee bend. (**d**) Four computer simulation models with 0°, 4°, 7°, 10° flexion of the femoral component against distal femoral anatomical axis and implant down-size model with 10° component flexion in Bi-Surface implant.

This study was performed using a musculoskeletal knee model in a computer simulation (LifeMOD/KneeSIM 2010; LifeModeler Inc., San Clemente, CA, USA). The simulation model consisted of a dynamic musculoskeletal program for knee modelling. The model included the tibiofemoral and patellofemoral contacts, lateral collateral ligament (LCL), medial collateral ligament (MCL), quadriceps muscle and tendon, patellar tendon, hamstring muscles, and elements of the knee capsule. All ligament bundles were modelled as nonlinear springs with material properties, as determined in a previous study^17^. The origins of the insertion points and stiffness were determined based on relevant anatomical studies^18–21^. The simulation program was previously validated to ensure appropriate estimates of kinematics, contact status, and contact force^22,23^. The computer simulation model with the attachments of ligaments, boundary conditions, and implants is shown in Fig. 1.

Based on poor clinical outcomes due to excessive flexion position of the femoral component in Bi-Surface Knee (Kyocera, Kyoto, Japan)^16^, the Bi-Surface Knee System with cruciate-substituting (CS) tibial insert were used for the computer simulations in this study. The Bi-Surface Knee System is a unique prosthesis with promising long-term durability and consists of a ball-socket joint as a third condyle, which allows contact between the femoral component and the polyethylene dish even in deep flexion^24^. NexGen LPS-flex (Zimmer Biomet Inc., Warsow, IN, USA), which is a fixed-bearing posterior-stabilised (PS) implant with a multi-radius femoral component, was also evaluated as one of the most widely used prosthesis^25^.

The three-dimensional bone model was constructed from whole-leg computed tomography (CT) images in one healthy volunteer (age: 30 years old, sex: male, height: 170 cm, weight: 80 kg, hip-knee-ankle angle: 0.1°varus, medial proximal tibial angle: 86.9°, mechanical lateral distal femoral angle: 87.0°, anterior bowing angle of the femur: 4.2°, posterior tibial slope: 4.1°) , and TKA with two types of implants was simulated. To be used as the standard model, the coronal alignment of the femoral component was set perpendicular to the coronal mechanical axis of the femur with a sulcus cut to determine the thickness of the distal femoral cut. An anterior reference was used to determine the antero-posterior position of the femoral component. The sagittal alignment was parallel to the distal femoral anatomical axis, and the anterior condyle of the femur was cut flush to the anterior border of the femoral cortex, with axial rotation parallel to the surgical epicondylar axis. The tibial component was placed perpendicular to the mechanical axis of the tibia for coronal alignment, preserving a native posterior slope (4°)^16^, and the rotation was parallel to Akagi’s line. Patella replacement was performed to maintain the original patellar thickness. An appropriately sized implant (femoral component size: Bi-Surface Knee; XLAG, LPS-flex; F) was placed in the computer simulation.

### Measurements

The computer simulation with the TKA prosthesis was used to simulate two cycles of squatting activity in a weight-bearing deep knee bend according to an Oxford-type knee rig (Fig. 1a,b). During the squatting activity, a constant vertical force was applied at the hip, corresponding to a body weight of 80 kg, which was converted to a ~ 4,000 N load on the knee. The knee model was flexed from full extension to 150° and then back to full extension within 4.5 s (Fig. 1c)^26^. During two cycles of the activity, the anteroposterior position of the facet centre of the medial and lateral compartment, the tibiofemoral intercomponent contact force of each condyle, patellofemoral contact force, collateral ligament forces, and quadricep muscle force were recorded. The values measured in the second squatting cycle were selected for the analyses because the first cycle was slightly unstable for fitting the bounding conditions of each intercomponent joint.

The experiments were performed by changing the sagittal alignment of the femoral component (Fig. 1d). First, the femoral component was rotated 4°, 7°, and 10° to flexion from the original position (0°) in the sagittal plane with an anterior reference. This rotation angle was selected based on the previous study in which the mean flexion angle of the femoral component was 4° with 3° of standard deviation, and patients with excessive flexion of the femoral component (≥ 8.5°) was inferior clinical outcomes^16^. The distal femoral cut was flexed from the original distal femoral anatomical axis, and the distal femoral cut was performed using a sulcus cut to preserve the extension gap. The most proximal point of the anterior flange was placed at the surface of the anterior femoral cortex to avoid notching or anterior overhang of the implant. The size of the implant was not changed, and the posterior overhang of the implant (6.7 mm and 6.3 mm increase in Bi-Surface Knee and LPS-flex, respectively, when flexing femoral component from 0° to 10°) was left. Secondly, the increased posterior overhang of the posterior condyle of the implant was reduced using an implant downsize (one size smaller) of a 10° flexion model.

## Results

**Figure 2:**
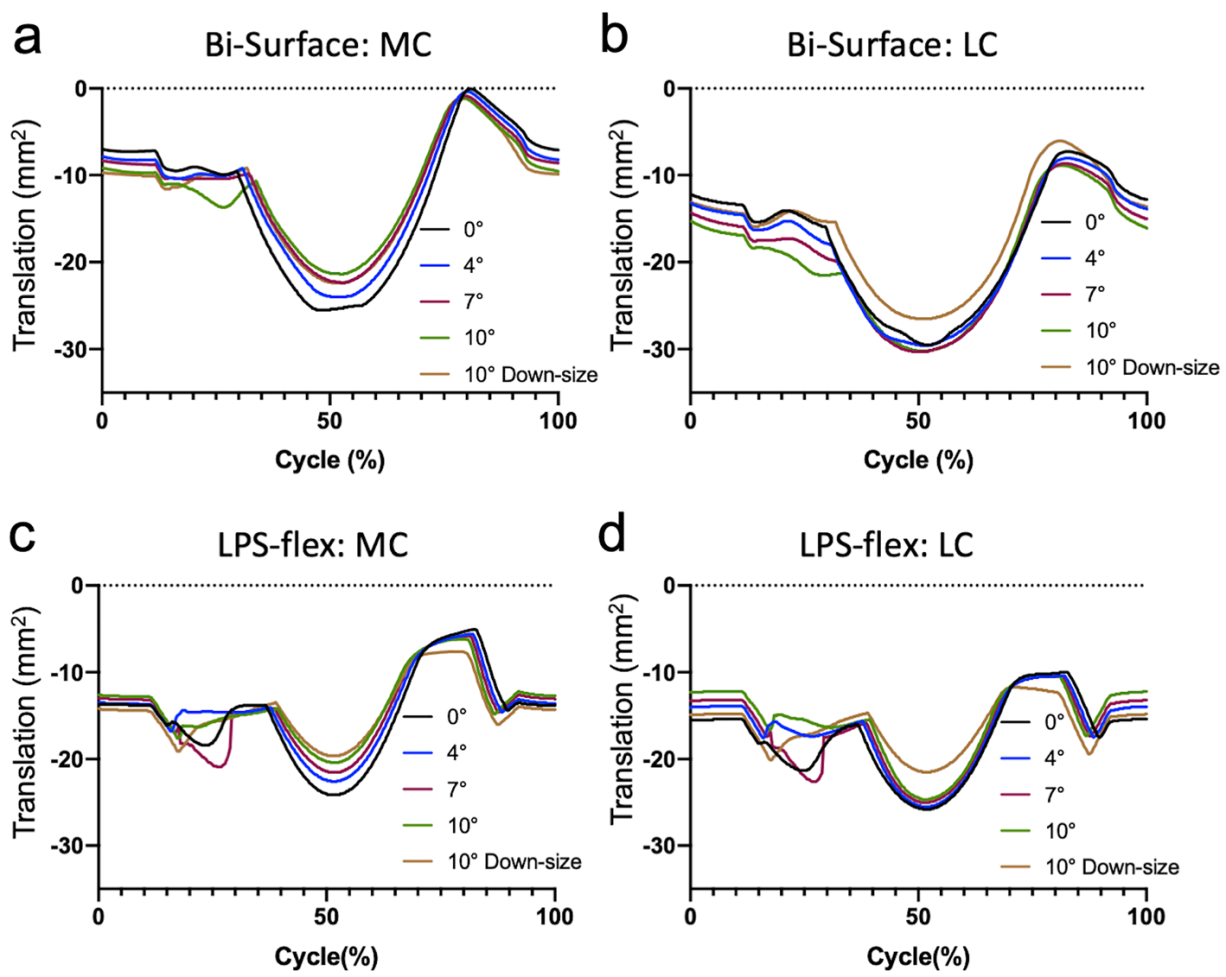
Antero-posterior translation of the facet centre of the Bi-Surface Knee (**a**: MC: medial compartment and **b**: LC lateral compartment) and the LPS-flex (**c**: MC, **d**: LC).

In the Bi-Surface Knee with 10° of flexion of the femoral component, gradual posterior translation was observed in both condyles during the 15% to 25% cycle (40° to 70° of knee flexion), followed by paradoxical anterior translation of the medial compartment (Fig. 2a). When the femoral component was downsized while maintaining a 10° flexion of the femoral component, this abnormal movement was not observed. Posterior translation of the lateral compartment was observed before bicondylar rollback by the flexion of the femoral component (Fig. 2b). In the LPS-flex, the 4° flexion model showed relatively stable medial compartment in the mid-flexion range (20% to 40% cycle), while other flexion angles showed paradoxical anterior translations (Fig. 2c,d). With an increase in the flexion of the femoral component, the medial compartment moved anteriorly during bi-condylar roll-back in both implants, but the lateral compartment did not show any remarkable changes (Fig. 2a–d). By downsizing the implant, the facet centre of the medial and lateral compartment shifted anteriorly during the roll-back and roll-forward phases (Fig. 2a–d).

**Figure 3:**
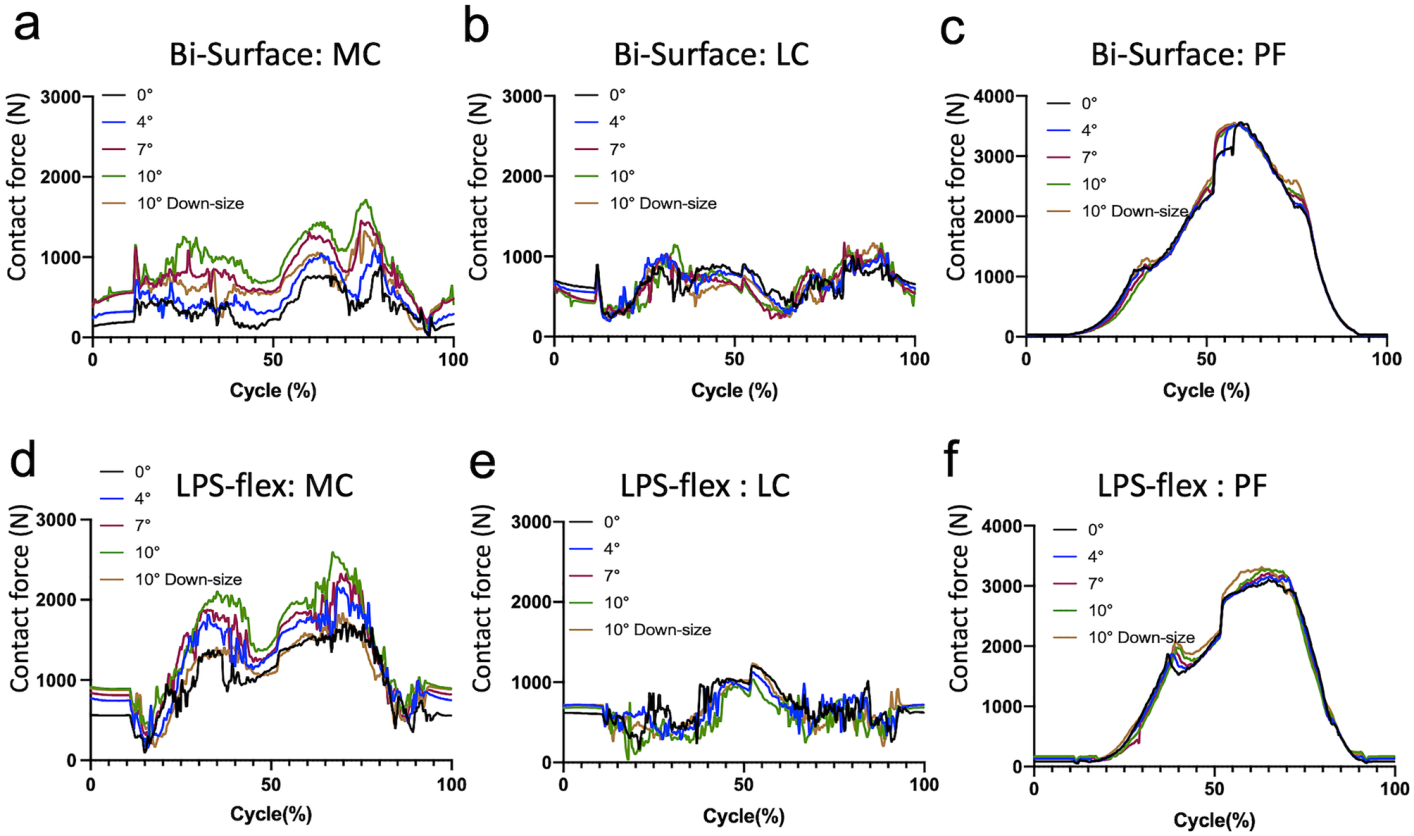
Contact force in the Bi-Surface Knee (**a**–**c**) and LPS-flex (**d**–**f**). (**a**,**d**) medial compartment, (**b**,**e**) lateral compartment, (**c**,**f**) patellofemoral joint. *MC* medial compartment, *LC* lateral compartment, *PF* patellofemoral joint.

In terms of the contact force of each compartment, the contact force of the medial compartment peaked in the extension phase (75% to 80% cycle, 75° to 50° of knee flexion in the Bi-Surface Knee and 65% to 75% cycle, 120° to 75° of knee flexion in the LPS-flex) (Fig. 3a,d). In both implants, the peak contact force of the medial compartment increased with the flexion of the femoral component, but the contact force in the lateral compartment did not show any remarkable changes (Fig. 3a,b,d,e, Tables 1 and 2). By downsizing the femoral component, the medial contact force was considerably reduced (Fig. 3a,d, Tables 1 and 2). The patellofemoral contact force was not greatly changed by flexion of the femoral component (Fig. 3c,f, Tables 1 and 2).

**Table 1:** Peak contact force of each compartment in Bi-Surface Knee.

Femoral component flexion angle	0°	4°	7°	10°	10° Down-size
Medial compartment (N)	895	1100	1460	1710	1330
Lateral compartment (N)	1010	1050	1180	1170	1170
Patellofemoral compartment (N)	3560	3520	3530	3520	3550

**Table 2:** Peak contact force of each compartment in LPS-flex.

Femoral component flexion angle	0°	4°	7°	10°	10° Down-size
Medial compartment (N)	1717	2166	2330	2604	1836
Lateral compartment (N)	1201	1123	1084	1035	1226
Patellofemoral compartment (N)	3102	3147	3208	3271	3307

**Figure 4:**
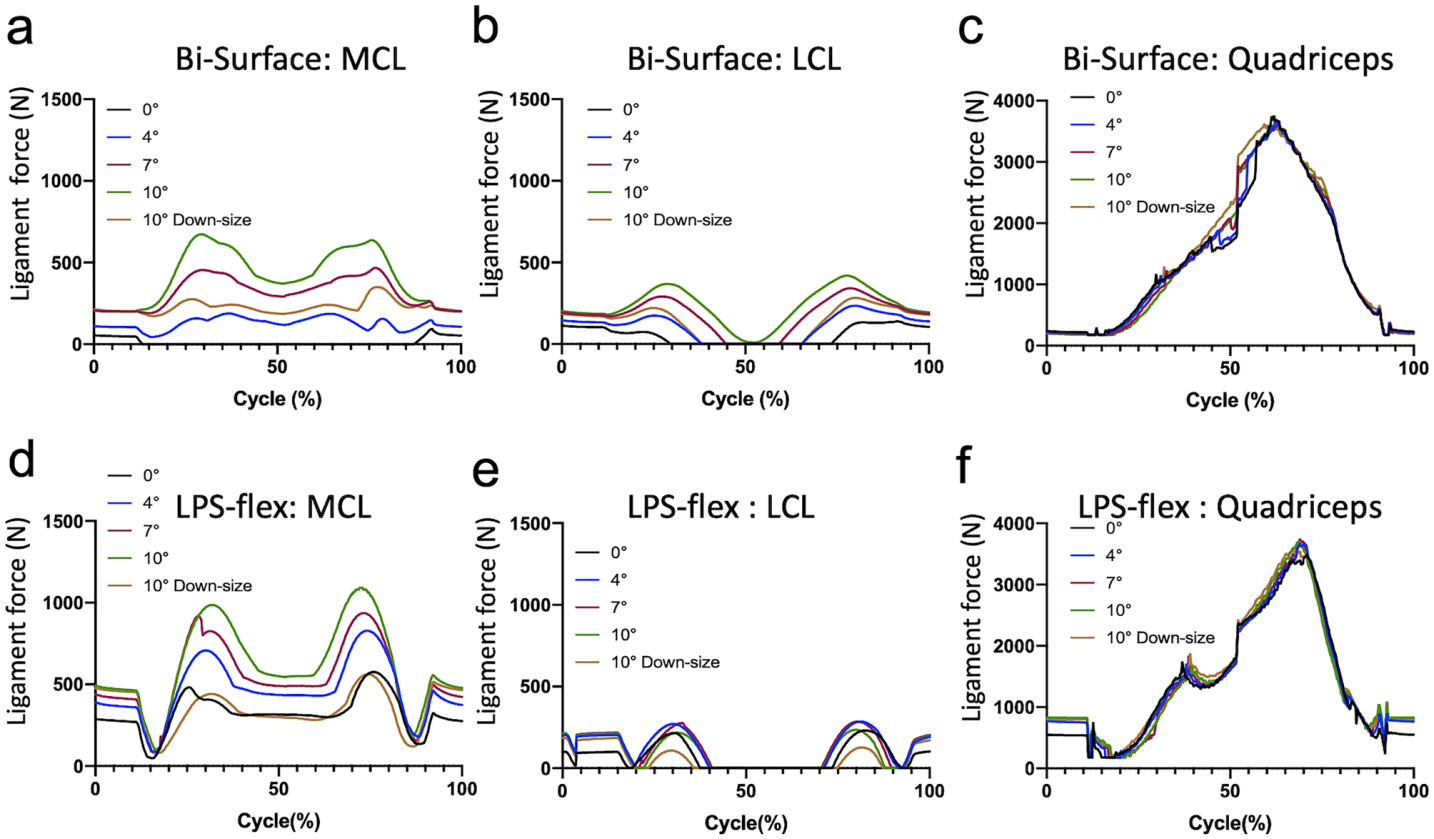
Ligament force in the Bi-Surface Knee (**a**–**c**) and LPS-flex (**d**–**f**). (**a**,**d**) medial collateral ligament, (**b**,**e**) lateral collateral ligament (**c**,**f**) quadriceps. *MCL* medial collateral ligament, *LCL* lateral collateral ligament.

When ligament force was observed, the MCL force increased as the flexion of the femoral component increased, and each peak was observed in both the flexion and extension phases (Fig. 4a,d). These peak MCL forces were observed at approximately 25% to 30% and 75% to 80% cycles corresponding to the mid-flexion range (approximately 50° to 80° of knee flexion). Flexion of the femoral component from 7° to 10° of knee flexion showed a considerable increase in the MCL force in both implants, which was remarkably reduced by the downsizing of the femoral component (52% reduction in the Bi-Surface and 48% reduction in the LPS-flex from the original size) (Fig. 4a,d, Tables 3 and 4). The effect on LCL was smaller than that on MCL, and the quadriceps force was not dramatically changed by the flexion of the femoral component or implant size (Fig. 4b,c,e,f, Tables 3 and 4).

**Table 3:** Peak ligament force of each ligament and quadriceps in Bi-Surface Knee.

Femoral component flexion angle	0°	4°	7°	10°	10° Down-size
Medial collateral ligament (N)	93	188	467	673	349
Lateral collateral ligament (N)	139	234	342	419	283
Quadriceps (N)	3740	3670	3650	3600	3620

**Table 4:** Peak ligament force of each ligament and quadriceps in LPS-flex.

Femoral component flexion angle	0°	4°	7°	10°	10° Down-size
Medial collateral ligament (N)	482	708	918	986	476
Lateral collateral ligament (N)	231	286	286	235	185
Quadriceps (N)	3492	3675	3736	3702	3570

## Discussion

This study demonstrated that excessive flexion of the femoral component increased the medial compartment contact force and MCL force in both CS and PS TKA. The Bi-Surface Knee showed mid-flexion instability which was represented by the paradoxical anterior translation when the implant was flexed at 10°. In the LPS-flex, a 4° flexion of the femoral component was most stabilised in mid-flexion, and other flexion angles showed paradoxical anterior translation. Taken together, slight flexion of the femoral component may be safe for knee biomechanics in these implants. Downsizing of the femoral component cancelled the increased contact and ligament forces, probably through decreased posterior overhang.

When each implant was compared, the Bi-surface Knee seemed more stable in mid-flexion range except 4°flexion model in which both implants showed good stabilization. Although the effect of flexion of the femoral component on contact and ligament force changes showed similar trends in both implants, the magnitude of peak contact force of Bi-Surface Knee was smaller than that of LPS-flex. In flexion range where LPS-flex showed peak contact force, a ball-socket joint in Bi-Surface knee had another contact area as a third condyle, which probably decrease the contact force of medial and lateral condyles. The differences in ligament forces in early to mid-flexion range was possibly due to the combined effect of stability, antero-posterior position, and rotation of the implant.

Previous studies have investigated the kinematic and biomechanical effects of flexion of the femoral component in TKA. A previous computer simulation study described that anteroposterior translation of the femoral component and quadriceps force decreased in both PS and cruciate-retaining TKA implants^8,15^ These studies evaluated a 10° range from − 3° of extension to 7° of flexion of the femoral component against the mechanical axis of the femur. In our study, the femoral component angle was defined against the distal femoral anatomical axis, which was 1.0° flexed to the mechanical axis of the femur in this case; the evaluation range was from 0° to 10° of flexion. Therefore, the investigated implant position was different from that used in previous studies. The other difference lies in the method of implant flexion. We flexed the femoral component with an anterior reference placed flush to the anterior cortex of the distal femur to avoid notch formation and anterior overhang of the femoral component. Thus, flexion of the femoral component resulted in an increase in the posterior overhang of the femoral component. In clinical reports, flexion of the femoral component and increased posterior condylar offset have been reported to be the result of posterior overhang of the femoral component^16,27^ However, in previous simulation articles, although the exact method of implant flexion was unclear, the femoral implant was flexed with a posterior reference, and anterior overhang of the anterior flange was observed^8,15^. In another biomechanical study using computer simulation, the effect of flexion and size of the implant was evaluated using cruciate-retaining TKA^28^ The evaluation range of flexion was 0° to 9° against the mechanical axis of the femur. In their study, the femoral component was also flexed with a posterior reference, and an increase of the posterior overhang was not created. The results showed that flexion of the femoral component increased the knee extensor moment arm in extension, reduced the quadriceps and patellofemoral contact forces, and provided stable kinematics. Downsizing of the femoral component shows mixed results, increasing patellofemoral contact force but decreasing medial patellofemoral ligament force and PCL force. The above-mentioned studies flexed the implant with a posterior reference, and flexion of the implant did not show a severe deteriorating effect on kinematics and biomechanics. In their model, anterior overhang of the anterior flange may exist, but posterior overhang was not observed. In contrast, in our study, the implant was flexed with anterior reference; therefore, increased posterior overhang caused a decrease in the flexion gap, leading to an increase in the joint/ligament force in the extensive flexion model.

The downsizing of the implant reduces the joint/ligament force without causing severe abnormal kinematics. When the posterior overhang was compared, the 4° flexion and 10° flexion downsize models had similar posterior overhangs. When the initial intramedullary guide was mistakenly inserted in the flexion position and anterior reference TKA was successfully performed by appropriate sizing of the posterior condyle, posterior overhang was avoided. This is the condition of the downsize model used in this study. However, posterior overhang could not be avoided when the implant was mistakenly flexed during implantation. In a previous study, 23% of undesired flexion of the femoral component was reported during the final implantation^29^. This error causes posterior overhang, which could result in excessive flexion in this study.

Clinically, the effects of flexion of the femoral component have been reported in various aspects. In terms of the longevity of the implant, flexion of the femoral component was one of the risk factors for revision TKA because anterior overhang of the anterior flange of the flexed implant might irritate the quadriceps tendon and cause patellar crepitus^30^. Another study reported that femoral implants with more than 3° of flexion have higher failure rates compared to neutral (0° to 3° of flexion) and extended femoral components^31^. As for the clinical outcomes, one study showed that patients with excessive flexion of the femoral component (> 8.5°) had inferior outcomes, although there was a wide safety range, which yielded good satisfaction and function^16^. Another study using a machine learning model prediction showed that the likelihood of being ‘satisfied or very satisfied’ and a knee ‘always feeling normal’ increased with a change in the tibial slope within 2° from the native slope and femoral component flexion of 0° to 7°^4^. It has been reported that although there is no consensus in the acceptable range of the sagittal alignment of the femoral component, excessive flexion should be avoided^5–8^. Currently, there is no definitive angle to illustrate ‘excessive’ flexion, but when observing the biomechanics of two types of implants, 10° of flexion to the distal femoral anatomical axis seemed to be an ‘excessive’ flexion, as it resulted in abnormal kinematics and excessive joint contact/ligament forces in both implants.

This study had several limitations. First, the simulation consisted of a virtual and variable model with a generally healthy knee joint consisting of interpolated material properties from cadaver studies. The kinematics and obtained forces have been validated for physiologically relevant motions of TKA, but the values obtained might not be entirely the same as in living patients with end-stage arthritis. Second, no statistical analysis was performed because a standard bone model was used in this study. Single healthy bone model which was validated with the real knee motion in fluoroscopic analysis was used in this study. Due to the ethical reason according to radiographic exposure, multiple bone models are currently unavailable. Changing the experimental conditions in a bone model, which was hard to be performed in the real world, is the strength of the computer simulation study, and numbers of studies have reported using single validated bone model^8,25,26,28,32–34^. However, there are anatomic variations depending on sex, race and individual even in healthy volunteer^35^. Progression of osteoarthritis possibly has additional anatomical change on femoral bowing, tibia vara, or tibial slope^36,37^. Preoperative and postoperative lower-leg alignment may affect the biomechanics after TKA^38^. However, only a neutral alignment bone model was simulated using a mechanical alignment TKA. Thus, further research on multiple bone models including an osteoarthritis knee would be ideal to obtain more representative information mimicking real-life clinical situations. Third, only two fixed-bearing implants, one CS and one PS implant were analysed in this study. It is unclear whether similar results would be obtained in cruciate-retaining or mobile-bearing TKA. Moreover, even within CS and PS TKA, optimal flexion of the femoral component may differ among prosthesis due to the difference in surface geometry. However, at least in the two implants evaluated in this study, excessive flexion of the femoral component was warned, as with poor outcomes in clinical studies^4,16^. Further studies with various types of implants would be ideal to generalise the effect of excessive flexion of the femoral component.

In conclusions, mild flexion of the 4° femoral component showed stabilised mid-flexion during deep knee bend activity, and the medial joint/ligament force increased as the flexion of the femoral component increased. As suggested by clinical studies, mild flexion of the femoral component is good for the target sagittal alignment of the femoral component, and excessive flexion should be avoided in light of knee biomechanics.

## Data Availability

The datasets used and/or analysed during the current study are available from the corresponding author on reasonable request.

## References

[1] Matsuda S, Kawahara S, Okazaki K, Tashiro Y, Iwamoto Y (2013). Postoperative alignment and ROM affect patient satisfaction after TKA. Clin. Orthop. Relat. Res..

[2] Grave PW (2022). Higher satisfaction after total knee arthroplasty using restricted inverse kinematic alignment compared to adjusted mechanical alignment. Knee Surg. Sports Traumatol. Arthrosc..

[3] Hamilton DF (2015). Implant design influences patient outcome after total knee arthroplasty: A prospective double-blind randomised controlled trial. Bone Joint J..

[4] Farooq H, Deckard ER, Arnold N, Meneghini RM (2021). Machine learning algorithms identify optimal sagittal component position in total knee arthroplasty. J. Arthroplasty.

[5] Gromov K, Korchi M, Thomsen MG, Husted H, Troelsen A (2014). What is the optimal alignment of the tibial and femoral components in knee arthroplasty?. Acta Orthop..

[6] Matziolis G, Krocker D, Weiss U, Tohtz S, Perka C (2007). A prospective, randomized study of computer-assisted and conventional total knee arthroplasty. Three-dimensional evaluation of implant alignment and rotation. J. Bone Joint Surg. Am..

[7] Minoda Y, Kobayashi A, Iwaki H, Ohashi H, Takaoka K (2009). TKA sagittal alignment with navigation systems and conventional techniques vary only a few degrees. Clin. Orthop. Relat. Res..

[8] Kang KT, Koh YG, Son J, Kwon OR, Park KK (2019). Flexed femoral component improves kinematics and biomechanical effect in posterior stabilized total knee arthroplasty. Knee Surg. Sports Traumatol. Arthrosc..

[9] Lu ZH (2012). Computed tomographic measurement of gender differences in bowing of the sagittal femoral shaft in persons older than 50 years. J. Arthroplasty.

[10] Chung BJ, Kang YG, Chang CB, Kim SJ, Kim TK (2009). Differences between sagittal femoral mechanical and distal reference axes should be considered in navigated TKA. Clin. Orthop. Relat. Res..

[11] Tsukeoka T, Lee TH (2012). Sagittal flexion of the femoral component affects flexion gap and sizing in total knee arthroplasty. J. Arthroplasty.

[12] Roßkopf J, Singh PK, Wolf P, Strauch M, Graichen H (2013). Influence of intentional femoral component flexion in navigated TKA on gap balance and sagittal anatomy. Knee Surg. Sports Traumatol. Arthrosc..

[13] Li G (2005). Anterior tibial post impingement in a posterior stabilized total knee arthroplasty. J. Orthop. Res..

[14] Collier MB, Engh CA, McAuley JP, Ginn SD, Engh GA (2005). Osteolysis after total knee arthroplasty: Influence of tibial baseplate surface finish and sterilization of polyethylene insert. Findings at five to ten years postoperatively. J. Bone Joint Surg. Am..

[15] Koh YG, Hong HT, Lee HY, Kim HJ, Kang KT (2021). Influence of variation in sagittal placement of the femoral component after cruciate-retaining total knee arthroplasty. J. Knee Surg..

[16] Nishitani K (2020). Excessive flexed position of the femoral component was associated with poor new Knee Society Score after total knee arthroplasty with the Bi-Surface knee prosthesis. Bone Joint J..

[17] Blankevoort L, Kuiper JH, Huiskes R, Grootenboer HJ (1991). Articular contact in a three-dimensional model of the knee. J. Biomech..

[18] Park SE (2005). The change in length of the medial and lateral collateral ligaments during in vivo knee flexion. Knee.

[19] LaPrade RF (2007). The anatomy of the medial part of the knee. J. Bone Joint Surg. Am..

[20] Wijdicks CA (2010). Structural properties of the primary medial knee ligaments. Am. J. Sports Med..

[21] Belvedere C (2012). Geometrical changes of knee ligaments and patellar tendon during passive flexion. J. Biomech..

[22] Okamoto S (2015). Effect of tibial posterior slope on knee kinematics, quadriceps force, and patellofemoral contact force after posterior-stabilized total knee arthroplasty. J. Arthroplasty.

[23] Tanaka Y (2016). How exactly can computer simulation predict the kinematics and contact status after TKA? Examination in individualized models. Clin. Biomech..

[24] Nakamura S (2017). Long-term durability of ceramic tri-condylar knee implants: A minimum 15-year follow-up. J. Arthroplasty.

[25] Watanabe M (2019). Impact of intraoperative adjustment method for increased flexion gap on knee kinematics after posterior cruciate ligament-sacrificing total knee arthroplasty. Clin. Biomech..

[26] Sekiguchi K (2019). Effect of tibial component alignment on knee kinematics and ligament tension in medial unicompartmental knee arthroplasty. Bone Joint Res..

[27] Ng N, Patton JT, Burnett R, Clement ND (2020). Sagittal alignment of the cemented femoral component in revision total knee arthroplasty influences the anterior and posterior condylar offset: Stem length does not affect these variables. Knee.

[28] Marra MA (2018). Flexing and downsizing the femoral component is not detrimental to patellofemoral biomechanics in posterior-referencing cruciate-retaining total knee arthroplasty. Knee Surg. Sports Traumatol. Arthrosc..

[29] Kuriyama S (2018). Bone-femoral component interface gap after sagittal mechanical axis alignment is filled with new bone after cementless total knee arthroplasty. Knee Surg. Sports Traumatol. Arthrosc..

[30] Dennis DA (2011). The John Insall Award: control-matched evaluation of painful patellar Crepitus after total knee arthroplasty. Clin. Orthop. Relat. Res..

[31] Kim YH, Park JW, Kim JS, Park SD (2014). The relationship between the survival of total knee arthroplasty and postoperative coronal, sagittal and rotational alignment of knee prosthesis. Int. Orthop..

[32] Mizu-uchi H (2022). Tibial sagittal and rotational alignment reduce patellofemoral stresses in posterior stabilized total knee arthroplasty. Sci. Rep..

[33] Nishitani K (2019). Valgus position of the femoral component causes abnormal kinematics in the presence of medial looseness in total knee arthroplasty: A computer simulation model of TKA for valgus knee osteoarthritis. Knee Surg. Sports Traumatol. Arthrosc..

[34] Kuriyama S, Ishikawa M, Furu M, Ito H, Matsuda S (2014). Malrotated tibial component increases medial collateral ligament tension in total knee arthroplasty. J. Orthop. Res..

[35] MacDessi SJ, Griffiths-Jones W, Harris IA, Bellemans J, Chen DB (2021). Coronal plane alignment of the knee (CPAK) classification. Bone Joint J..

[36] Higano Y (2016). The varus alignment and morphologic alterations of proximal tibia affect the onset of medial knee osteoarthritis in rural Japanese women: Case control study from the longitudinal evaluation of Matsudai Knee Osteoarthritis Survey. J. Orthop. Sci..

[37] Umatani N (2023). Femoral bowing affects varus femoral alignment but not patient satisfaction in mechanically aligned total knee arthroplasty. Eur. J. Orthop. Surg. Traumatol..

[38] Song Y (2021). Biomechanical comparison of kinematic and mechanical knee alignment techniques in a computer simulation medial pivot total knee arthroplasty model. J. Knee Surg..

